# Double Meniscal Ossicle, the First Description: CT and MRI Findings—Different Etiologies

**DOI:** 10.1155/2015/737506

**Published:** 2015-12-15

**Authors:** Puneeth Kumar, Amit Kumar Dey, Kartik Mittal, Rajaram Sharma, Priya Hira

**Affiliations:** Department of Radiology, Seth G. S. Medical College and KEM Hospital, Mumbai 400012, India

## Abstract

We present a case of 2 ossicles in the medial meniscus with emphasis on MRI and CT findings. Meniscal ossicle is a rare entity and is quite uncommon on the medial side. By showing the typical signal characteristics and intrameniscal location, MRI can be helpful in distinguishing this from other more clinically significant abnormalities. It should be kept as differential from synovial chondromatosis or sesamoid bones like fabella as management is different for all of these entities.

## 1. Introduction

Ossicles within the meniscus of the knee are reported as a rare finding [1]. The ossicle could be described as corticocancellous bones with central fatty marrow completely surrounded by the meniscal fibrocartilage. They are usually symptomatic and discovered on knee radiographs [1–4]. Incidentally, they can occur in asymptomatic person. Radiological differentiation can be made from osteochondral loose bodies and chondrocalcinosis by its ossified appearance and its typical location within the meniscus. Correct diagnosis is required so that unnecessary surgery is avoided and protracted search of free fragment is not carried out [5, 6]. We present a case of 2 ossicles in the medial meniscus with emphasis on MRI and CT findings.

## 2. Case Report

**Figure 1:**
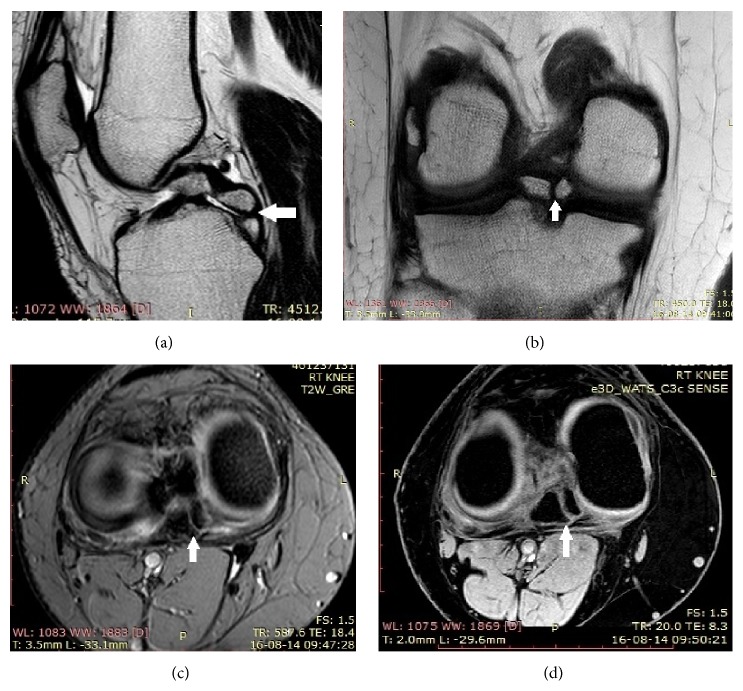
A 21-year young male student presented with chronic pain in the right knee joint for 1-2 years subsequently diagnosed as double meniscal ossicle of the knee. (a) Sagittal T1W image shows two small lesions (solid arrow), isointense to bone marrow in relation to the posterior horn of the medial meniscus, with a hypointense rim. (b) Coronal T1 W images confirm the isointensity of the lesions (solid arrow) to the bone marrow in relation to posterior aspect of medial meniscus. (c) Axial high-resolution T2 GRE image confirms the relationship of the meniscal ossicle (solid arrow) with the posterior horn of the medial meniscus. (d) Axial high-resolution T2 DESS image confirms the relationship of the meniscal ossicle (solid arrow) with the posterior horn of the medial meniscus.

**Figure 2:**
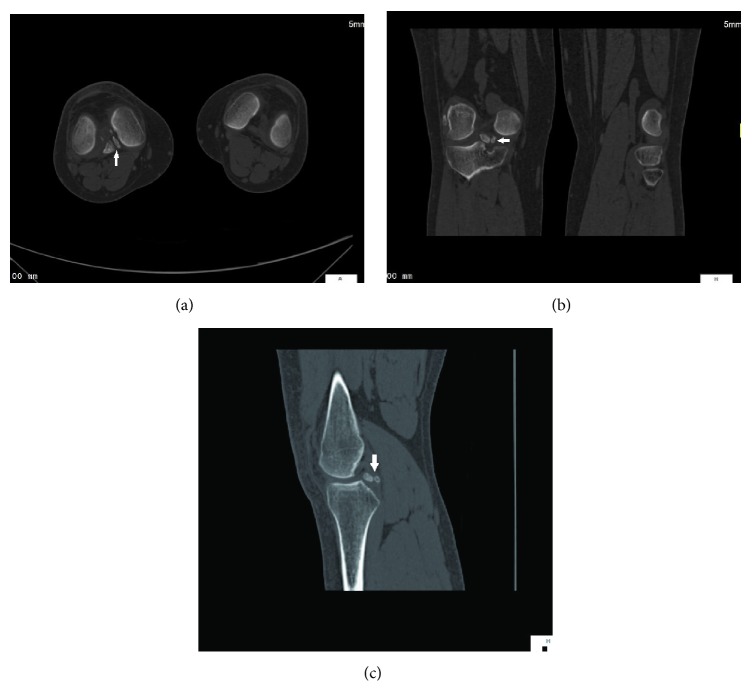
A 21-year young male student presented with chronic pain in the right knee joint for 1-2 years subsequently diagnosed as double meniscal ossicle of the knee. (a) Plain CT axial bone window images show well defined lamellated bone density lesions, two in number (solid arrow) in intra-articular region on medial aspect of right knee. (b) Plain CT coronal bone window images show well defined lamellated bone density lesions, two in number (solid arrow) in intra-articular region on medial aspect of right knee. (c) Plain CT sagittal bone window images show well defined lamellated bone density lesions, two in number (solid arrow) in intra-articular region on medial aspect of right knee.

A 21-year young male student presented with chronic pain in the right knee joint for 1-2 years. There was no recent history of trauma. There was not any other relevant past history. On clinical examination, there was mild swelling without any restriction of movements. Posterior drawer sign and the posterior tibial sag sign were negative. There was not any history of sudden violent trauma like dashboard injury. So PCL avulsion was ruled out. The radiographs were unavailable but were reported to be normal. Patient was sent for further investigations in the form of MRI. MRI showed two small lesions isointense to bone marrow in relation to the posterior horn of the medial meniscus, with a hypointense rim suggestive of meniscal ossicle (Figures 1(a), 1(b), 1(c), and 1(d)). It was confirmed on plain CT axial, coronal, and sagittal bone window images which showed well defined lamellated bone density lesions, two in number in intra-articular region on medial aspect of right knee (Figures 2(a), 2(b), and 2(c)). Patient was put on analgesics and advised to take rest but did not improve. Arthroscopic findings of the patient confirmed our findings and avulsion of the PCL was ruled out. On entering the joint, a bulge in posterior horn of medial meniscus was seen and the rest of the joint appeared normal. PCL was normal with no signs of avulsion. So he was operated and on follow-up pain has subsided and patient is doing well. The surface of the excised bony fragment was noncystic, hard, and not irregular as usually found in the case of the ossicle.

## 3. Discussion

In 1934, the first case of meniscal ossicle was reported by Burrows [7]. To the best of our knowledge, in these 70 odd years, it has been reported 41 times [8]. It is not clear whether this is because it is an underdiagnosed/underreported condition or because it is actually an uncommon occurrence. Different theories are proposed for the etiology of the meniscal ossicle. Firstly, some consider it to be a degenerative phenomenon where areas of mucoid degeneration are replaced by bone [9]. Secondly some suggest it as posttraumatic sequelae with development of heterotopic ossification [2, 5–7]. Third theory proposes it to be a vestigial structure based on its presence in animal species like domestic cats, rodents, and Bengal tigers [1, 5]. The last theory suggests meniscal ossicles as bone fragments coming from the tibia at meniscal root insertion sites [8, 10]. The normal contour of the adjoining bone on MRI however, as in this case, argues against the last theory. In short, there is no definite consensus on the etiology of meniscal ossicles. Most patients complain of intermittent pain; however, since many patients also have other associated abnormalities, the relationship between the ossicles and pain is not definite [8]. A locking sensation is usually not experienced as would be expected with a free intra-articular body [8]. Most cases describe meniscal ossicles in the posterior horn of medial meniscus and very rarely in the anterior horn of the lateral meniscus [8]. It is important to differentiate meniscal ossicle from osteochondral loose bodies and chondrocalcinosis. Osteochondral loose bodies can easily be differentiated from ossicle because of defect in the articular cartilage of distal femur [11]. Loose body is frequently found lying in the superolateral part of the anterior compartment of the knee and is composed predominantly of calcified cartilage and subchondral bone [11]. When there is confusion to differentiate loose bodies from meniscal ossicle on plain radiographs, a meniscal ossicle can be differentiated by its MRI characteristics which include an intrameniscal location, internal signal intensity of marrow, and a surrounding rim of low signal intensity corresponding to cortex [12]. Chondrocalcinosis may create calcific density within the body of meniscus; it is typically punctuated and linearly arranged; it will not have well defined cancellous bone of meniscal ossicle [11].

## 4. Conclusion

Meniscal ossicle is a rare entity and is quite uncommon on the medial side. By showing the typical signal characteristics and intrameniscal location, MRI can be helpful in distinguishing this from other more clinically significant abnormalities. It should be kept as a differential from synovial chondromatosis or sesamoid bones like fabella as management is different for all of these entities.
